# Establishment and Validation of a Comprehensive Prognostic Model for Patients With HNSCC Metastasis

**DOI:** 10.3389/fgene.2021.685104

**Published:** 2021-07-12

**Authors:** Yajun Shen, Lingyu Li, Yunping Lu, Min Zhang, Xin Huang, Xiaofei Tang

**Affiliations:** ^1^Division of Oral Pathology, Beijing Institute of Dental Research, Beijing Stomatological Hospital, Capital Medical University, Beijing, China; ^2^Department of Oral and Maxillofacial Surgery, Beijing Stomatological Hospital, Capital Medical University, Beijing, China

**Keywords:** head and neck squamous cell carcinoma, metastasis, overall survival, prognostic signature, nomogram

## Abstract

**Objective:**

To identify biomarkers related to head and neck squamous cell carcinoma (HNSCC) metastasis and establish a prognostic model for patients with HNSCC.

**Methods:**

HNSCC mRNA expression data of metastasis and non-metastatic samples were downloaded from The Cancer Genome Atlas (TCGA) and Gene Expression Omnibus (GEO) databases. After screening the differentially expressed genes (DEGs) in the two datasets, a prognostic model, including clinical factors and biomarkers, was established, and verified in 36 samples of HNSCC by quantitative real-time transcription (qRT)-PCR. Gene Ontology (GO), Kyoto Encyclopedia of Genes and Genomes (KEGG), and Gene sets enrichment analysis (GSEA) were consulted to explore the functions of the DEGs.

**Results:**

In total, 108 DEGs were identified. GSEA, GO, and KEGG analyses showed that these DEGs were mainly involved in the proliferation and metastasis of HNSCC. Six genes that were significantly related to metastasis, immune cell infiltration and prognosis were further identified to construct a prognostic gene signature. The reliability of the gene signature was verified in 36 samples of HNSCC. A prognostic model, including tumor stage, risk level, and a nomogram for prediction were further established. Receiver operating characteristic (ROC) analysis, decision curve analysis (DCA), C-index, and calibration plots showed that the model and nomogram perform well.

**Conclusion:**

We constructed a six-gene signature and a nomogram with high performance in predicting the prognosis of patients with HNSCC metastasis.

## Introduction

Head and neck cancers (HNCs) rank sixth among the most common cancers worldwide. They arise anywhere in the head and neck, including the tongue, palate, buccal mucosa, throat, and pharynx (Marur and Forastiere, 2016). According to their pathological classification, the most common type is head and neck squamous cell carcinoma (HNSCC), accounting for 95% of HNCs. Continuous exposure to tobacco or alcohol are the main risk factors for HNSCC, and HPV infection is the most important risk factor for oropharyngeal tumors (Hatcher et al., 2016; Lydiatt et al., 2017). Although there are some ways to treat HNSCC, the survival rate of patients is still very low and the 5-year survival rate has remained below 60% in the past few decades (Miller et al., 2016). The main reasons for death are invasion and metastasis (Zhang et al., 2018). Due to the rich lymphatic system in the head and neck, lymphatic metastasis often occurs in HNSCC, and distant metastasis is also likely (Cho et al., 2015; Duprez et al., 2017). Therefore, the clinical staging of HNC is mainly based on the primary site of the tumor (T), the number of lymph nodes involved (N), and the presence of distant metastasis (M). TNM classification plays an important role in diagnosis, clinical treatment, and cancer registry activities (Huang and O’Sullivan, 2017). However, the tumor staging system is only based on clinicopathological data, and the key biomarkers and exact targets for predicting the development and prognosis of HNSCC remain unavailable. Currently, second-generation gene sequencing has brought a promising future for identifying valuable prognostic factors in HNSCC (Kamps et al., 2017). Although some biomarkers for HNSCC have been developed based on second-generation sequencing, there seems to be no perfect biomarkers that can predict the prognosis of HNSCC patients with metastasis.

The latest progress in the development of whole-genome sequencing and bioinformatics technology has provided new highlights for cancer genomes. The common open tumor database includes The Gene Expression Omnibus (GEO) and The Cancer Genome Atlas (TCGA), which use innovative genome analysis technologies to accelerate the comprehensive understanding of cancer genetics, thereby helping to develop new strategies for cancer diagnosis, prevention and treatment (Zhang Z. et al., 2019).

**FIGURE 1:**
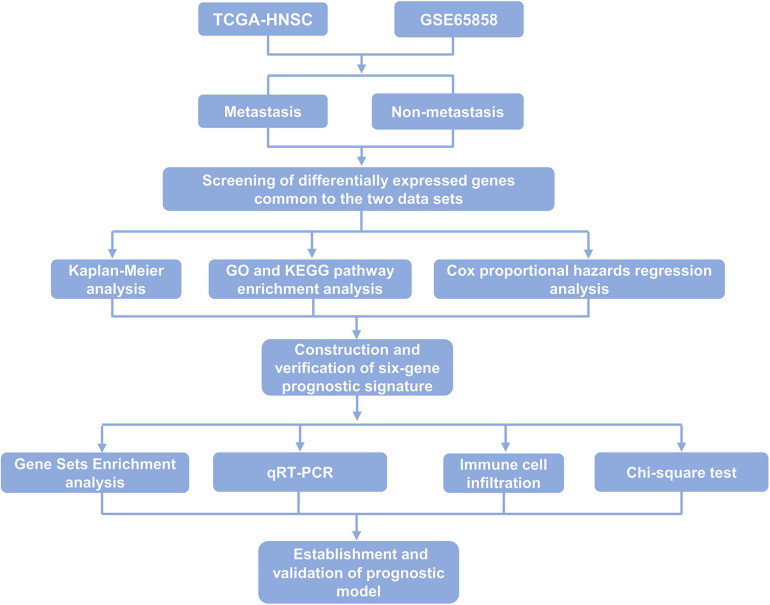
Flow chart of the main procedures of this study.

In this study, the mRNA expression profiles and metastasis-related clinical data of 499 and 270 HNSCC samples were obtained from the TCGA database and GEO database, respectively. Six key genes related to metastasis of HNSCC were screened out, namely, SYT14, METTL7B, FOXA2, GNG8, TNFRSF13B, and MYO1H, and the expression of these six genes was further verified in 36 samples of HNSCC patients. A novel multi-factor prognostic model, which included tumor stage and risk level was established and could predict the prognosis of patients with HNSCC effectively. The main design and process of this study are shown in Figure 1.

## Materials and Methods

### Data Source

The mRNA expression data and clinical data of 528 HNSCC samples were obtained from the TCGA dataset^1^ and 270 HNSCC samples from GEO datasets^2^, respectively, the accession number of GEO datasets is GSE65858. The following inclusion criteria were used: (1) Patients pathologically diagnosed with HNSCC, with no history of tumors in any other part; (2) Patients with complete clinical data and follow-up data; (3) Patients whose survival time was more than 1 month. Since these databases are public, ethical reviews were exempt. In this study, 499 HNSCC samples from TCGA and 270 HNSCC samples from GSE65858 were enrolled for further analysis. In addition, genes with lower expression levels were deleted. As R package SVA can estimate surrogate variables, directly adjust the known batch effects, and adjust the batch and latent variables in the prediction problem, SVA was used to eliminate batch effects and bias between different data sets (Leek et al., 2012). Moreover, the RNA-seq data of the two data sets were converted into transcripts per million (TPM) values and then transformed to log2 format, the “scale” function in the R package limma was further used for normalization. these samples were divided into two groups according to N stage and M stage. N0 and M0 samples were all pooled in the non-metastatic group, while the others were classified as the metastatic group.

### Gene Set Enrichment Analysis (GSEA)

To identify the KEGG pathway enrichment between non-metastatic group and metastatic group, the RNA expression data of the metastatic group and the non-metastatic group were extracted from the TCGA data set, GSEA^3^ was performed using GSEA software (version 4.1.0). the predefined gene set “c2.cp.kegg.v7.2.symbols.gmt” was downloaded from the Molecular Signatures Database (MSigDB). Pathways with *FDR* < 0.05 after performing 1,000 permutations were considered to be significantly enriched.

### Analysis of Differentially Expressed Genes

R package edgeR was used to identify differentially expressed genes (DEGs) between non-metastasis group and metastasis group with a cutoff of *FDR* < 0.05, and | log_2_FoldChange| < 0.5 in TCGA and GSE65858. The intersection function in R Studio was applied to determine the common DEGs between GSE65858 and TCGA. In order to explore the functions of these DEGs, David was used to perform GO and KEGG pathway enrichment analysis. *P* < 0.05 was defined as significant enrichment.

### Kaplan-Meier (KM) Survival Analysis of DEGs

X-tile software (Version 3.6.1) was used to define the best cut-off value for each DEGs, the 499 HNSCC samples in TCGA were divided into high-expression group and low-expression group according to the cut-off value. The KM method in the survival R package was used to found the DEGs that had a significant impact on the overall survival rate, two-stage method were applied to test the significance of the survival analysis. In the first stage, the log-rank test was performed, if *P* < 0.05 was obtained, the entire testing procedure ended, and the two hazard rates were considered to be significantly different. If the two hazard rates crossed, the stage two would be performed by landmark test to calculate the significance before and after the cross point (Qiu and Sheng, 2008).

### Identification of DEGs Related to Prognosis

To further identify the DEGs related to prognosis, univariate Cox proportional hazards regression analysis was utilized to analyze the degree of risk of the DEGs selected above. Then, the genes with *P* < 0.05 were included to multivariate Cox proportional hazards regression analysis to find a panel of key candidate genes significantly related to metastasis and prognosis. The best panel was identified by adopting a selection strategy based on the Akaike information criterion (AIC); the less information lost, the higher the quality of the model. GSEA and predefined gene set “c2.cp.kegg.v7.2.symbols.gmt” were used again to explore the functional pathways in which the high and low expression of these candidate genes were significantly enriched (*FDR* < *0.05*).

### Construction and Validation of the Multi-Gene Prognostic Signature

Next, a multi-gene prognostic signature was constructed with the candidate genes for HNSCC. All the HNSCC samples were scored using the following equation: Risk score = Σexp (RNAi) × coef (RNAi); where exp (RNA) is the expression level of RNA, and the coefficient (RNA) is the regression coefficient calculated by the multivariate Cox proportional hazards regression model. Then, according to the risk score, the 499 HNSCC samples in TCGA were divided into two groups by X-tile: low-risk and high-risk groups. KM survival analysis and log-rank test were performed between the two groups. In addition, the accuracy of the risk score model for predicting prognosis was assessed using the ROC curve and the area under the curve (AUC) was calculated. GSE65858 was used as an external validation of the risk model.

### Patients and Tissue Samples

A total of 36 HNSCC samples were obtained from Beijing Stomatological Hospital of Capital Medical University. This study complied with the Declaration of Helsinki and was approved by the Research Ethics Committee of Beijing Stomatological Hospital of Capital Medical University. Patients were selected according to the following criteria: (1) Patients with HNSCC pathologically diagnosed without other tumors and tumor history; (2) Patients with HNSCC who underwent primary tumor resection and neck lymphatic dissection, but did not receive radiotherapy and/or chemotherapy; (3) Patients with complete follow-up data. The HNSCC tissues obtained during the operation was immediately frozen in liquid nitrogen for storage. As all patients were in M0 stage, patients in N0 stage were defined as non-metastatic group, and patients with N1, N2, and N3 stages were defined as metastatic group.

### Quantitative Real-Time Transcription (qRT)-PCR

To further verify the accuracy of the multi-gene signature, total RNA was extracted from all the HNSCC tissues using TRIzol (Invitrogen Life Technologies, United States) reagent according to the manufacturer’s instructions. cDNA was synthesized using a High-Capacity cDNA Reverse Transcription Kit (Applied Biosystems, United States). GAPDH was used as an internal control, and mRNA expression levels were determined by qRT-PCR using SYBR Green (Qiagen, Germany). Gene expression was calculated using the 2^–△△CT^ method. All primers, listed in Table 1, were designed and compounded by Sangon Biotech (Shanghai, China).

**TABLE 1:** The primers used in qRT-PCR.

Gene name	Primer	Sequence (5′–3′)
GNG8	Forward	GAACATCGACCGCATGAAGGT
	Reverse	AGAACACAAAAGAGGCGCTTG
MYO1H	Forward	TAGCCCGTGACAGACTGCT
	Reverse	TTGGTAGACAACTCGGGACTT
TNFRSF13B	Forward	GAGCAAGGCAAGTTCTATGACC
	Reverse	CCTTCCCGAGTTGTCTGAATTG
METTL7B	Forward	TGCTCTTTTTCTGGGAGCAT
	Reverse	GCTGTCGTTCCATTTGGATT
SYT14	Forward	GGTGGAGAGAGAACCTGTGG
	Reverse	ATCTGGAAACCCGCCAACAT
FOXA2	Forward	CGTCGCTGGCTGGCATGTC
	Reverse	GGCTCAGACTCGGACTCAGGTG
GAPDH	Forward	GGAGAAACCTGCCAAGTATGA
	Reverse	CAACCTGGTCCTCAGTGTAGC

### Chi-Square Test

Chi-square test was performed to further explore which clinical factors were significantly related to risk level and survival status in TCGA, such as age, sex, smoking and drinking habits, location of tumor, HPV status, Pathological grade, TNM stage, *P* < 0.05 was considered to be significantly correlated.

### The Correlation Between the Risk Level and Immune Cell Infiltration

The R package CIBERSORT was applied in TCGA and GSE65858 to quantify the proportions of 22 immune cell types in the tumor microenvironment, and to find immune cells that are significantly related to the risk level (*P* < 0.05). The immune scores of 499 samples in TCGA were calculated by the ESTIMATE algorithm.

### Establishment and Validation of the Multi-Factor Prognostic Model

To establish an efficient multi-factor prognostic model, the 499 samples in TCGA were randomly assigned to two cohorts: the training cohort (*n* = 250) for the establishment of the model and the testing cohort (*n* = 249) for internal validation, GSE65858 was used as an external validation again. The risk level and clinical factors of the training cohort, including age, gender, smoking, drinking, location of tumor, HPV status, pathological grade and TNM stage, were included in the univariate Cox proportional hazards regression analysis. Next, we conducted multivariate Cox proportional hazards regression analysis on the factors with *P* < 0.05. In the output results, *P* < 0.05 was considered as an independent predictor of prognosis. Then, a multi-factor prognostic model was constructed based on the results of the Cox proportional hazards regression analysis. A nomogram was used to estimate the 1-, 3-, and 5-year survival rates of patients with HNSCC. Calibration plots and ROC curves were constructed to assess the predictive accuracy of this nomogram in predicting prognosis. Decision curve analysis (DCA) was performed to further evaluate the clinical net benefit of the nomogram. All the analyses were also applied to the internal validation cohort and external validation cohort. The C-index values of the nomogram in the three cohorts were calculated.

## Results

### DEGs Identification

**FIGURE 2:**
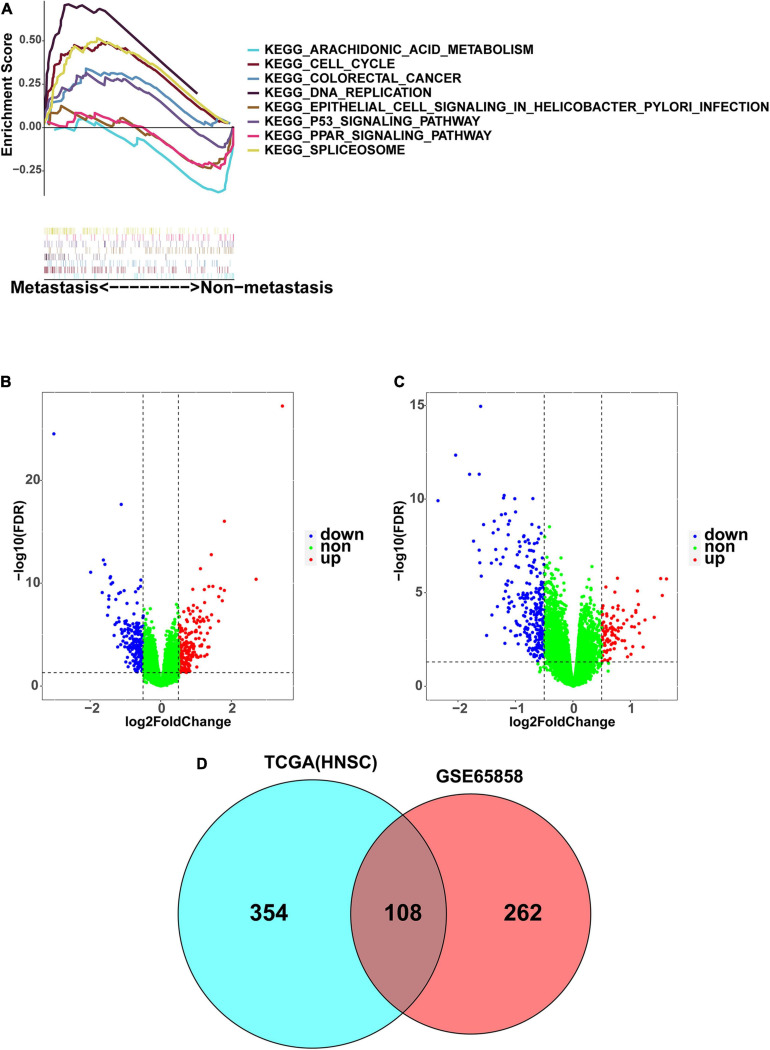
Identification of DEGs associated with HNSCC metastasis. **(A)** The GSEA enrichment analysis between metastasis group and non-metastasis group. **(B)** The volcano map presenting the DEGs involved in metastasis in TCGA-HNSC. **(C)** The volcano map presenting the DEGs involved in metastasis in GSE65858. Blue: downregulated; Red: upregulated. **(D)** As shown in Venn diagram, there are 108 DEGs shared by GSE65858 (red) and TCGA-HNSC (blue).

In the TCGA dataset, the 499 HNSCC samples were divided into a non-metastatic group of 163 samples and a metastatic group of 336 samples. In the GSE65858 dataset, the 270 HNSCC samples were divided into a non-metastatic group of 93 samples and a metastatic group of 177 samples. To explore the potential difference between the two groups in regard to the expression of genes involved in the KEGG pathway, GSEA analysis was conducted. The results showed that the metastasis group was mainly enriched in DNA replication, cell cycle, spliceosome, the P53 signaling pathway, and colorectal cancer, while the non-metastasis group was mainly enriched in arachidonic acid metabolism, the PPAR signaling pathway, and epithelial cell signaling in Helicobacter pylori infection (Figure 2A). We then found 462 DEGs in the TCGA dataset (Figure 2B), and 370 DEGs in the GSE65858 dataset (Figure 2C). There were 108 common DEGs, of which 69 were down-regulated and 39 were up-regulated (Figure 2D).

### Functional Enrichment Analysis of DEGs

**FIGURE 3:**
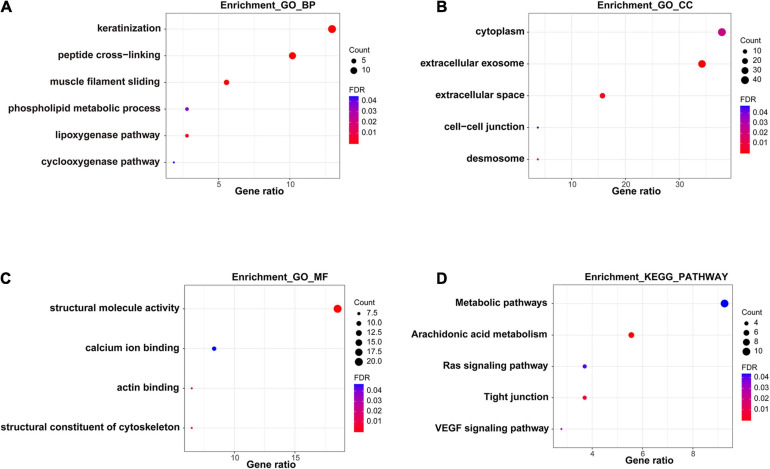
Functional enrichment analysis of DEGs. **(A–C)** GO analysis of DEGs. **(D)** KEGG pathway analysis of DEGs.

In order to further explore the functions and pathways of these 108 DEGs, we performed GO and KEGG enrichment analysis. A number of GO terms and pathways related to tumors were enriched, mainly extracellular exosome, muscle filament sliding, keratinization, metabolic pathway, the Ras signaling pathway, arachidonic acid metabolism, and the VEGF signaling pathway (Figure 3).

### KM Survival Analysis of DEGs

**FIGURE 4:**
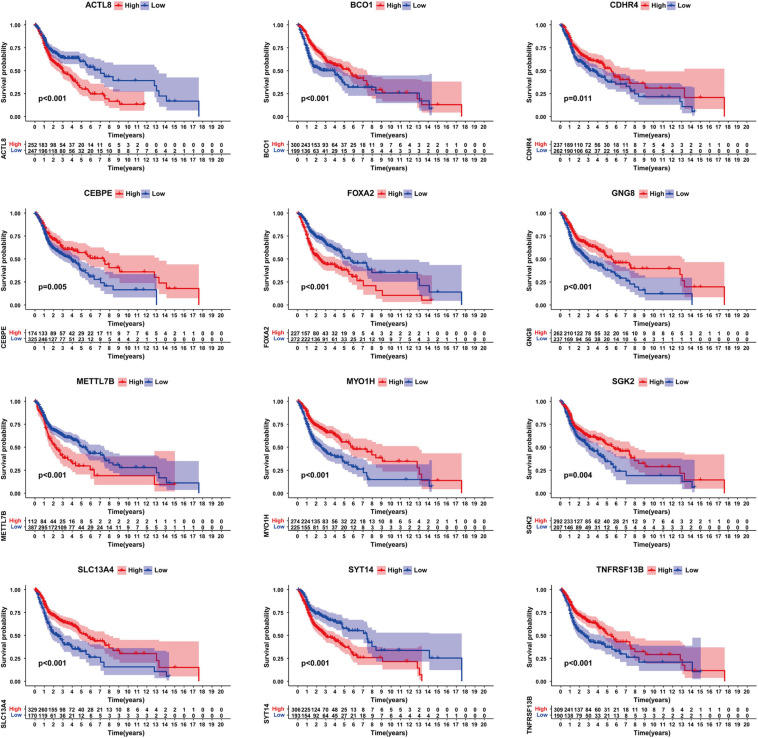
Survival analysis of genes significantly related to survival. Including ACTL8, BCO1, CDHR4, CEBPE, FOXA2, GNG8, METTL7B, MYO1H, SGK2, SLC13A4, SYT14, and TNFRSF13B.

Next, we organized the clinical data of HNSCC samples in the TCGA database and used the KM method to further obtain the DEGs that may influence survival outcomes. We found that ACTL8, BCO1, CDHR4, CEBPE, FOXA2, GNG8, METTL7B, MYO1H, SGK2, SLC13A4, SYT14, and TNFRSF13B had a significant impact on the overall survival rate (*P* < 0.05) (Figure 4).

### Construction and Validation of Prognostic Gene Signature

**FIGURE 5:**
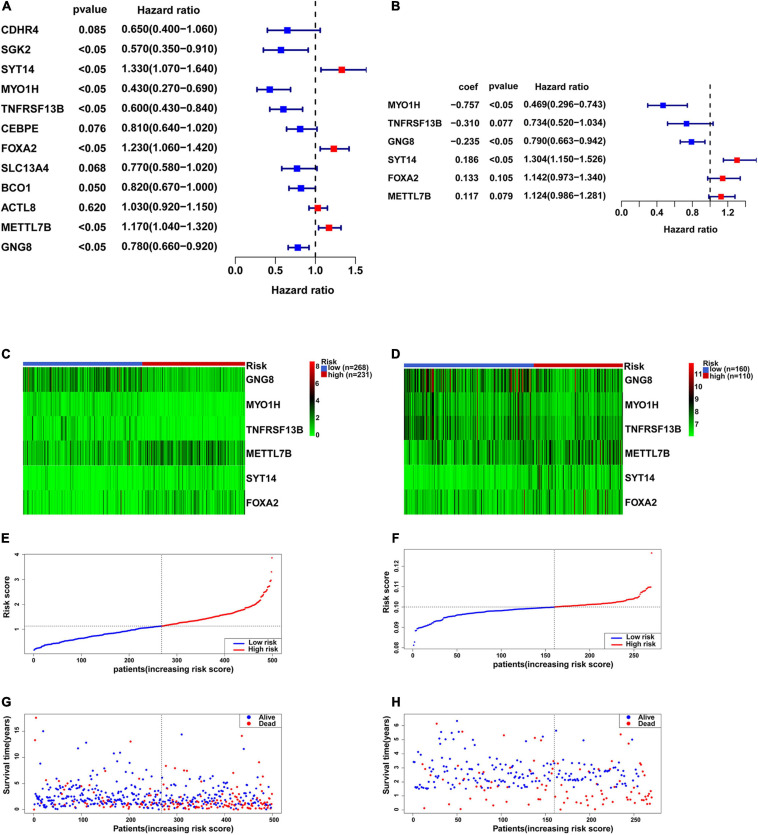
Construction of the six-gene prognostic signature. **(A)** Univariate Cox proportional hazards regression analysis was used to assess whether genes have significant prognostic value. **(B)** Multivariate Cox proportional hazards regression analysis was used to construct a risk biological prognostic signature. coef is the regression coefficient. **(C)** Heatmap of the six genes in the signature in TCGA. **(D)** Heatmap of the six genes in the signature in GSE65858. **(E)** The risk score of each HNSCC patient in TCGA. **(F)** The risk score of each HNSCC patient in GSE65858. **(G)** The survival status and overall survival time of each HNSCC patient in TCGA. **(H)** The survival status and overall survival time of each HNSCC patient in GSE65858.

Univariate Cox proportional hazards regression analysis was performed with the 12 candidate genes, and seven genes among them were obtained that were significantly associated with the outcome of each patient (*P* < 0.05), including four low-risk genes (HR < 1): SGK2, MYO1H, TNFRSF13B, and GNG8, as well as three high-risk genes (HR > 1): FOXA2, METTL7B, and SYT14 (Figure 5A). The above seven genes were included into multivariate Cox proportional hazards regression analysis. Finally, we obtained a panel of genes with the lowest AIC values, including MYO1H, TNFRSF13B, GNG8, FOXA2, METTL7B, and SYT14. The *P*-values of TNFRSF13B, FOXA2, and METTL7B were greater than 0.05, indicating that TNFRSF13B, FOXA2, and METTL7B could not be used as independent prognostic factors, but could be treated as auxiliary prognostic factors, so they were also retained (Figure 5B). It was worth noting that GNG8 and TNFRSF13B were highly expressed in the metastasis group, but played an opposite role in the prognosis (Figures 5C,D). A prognostic signature containing these six genes was established to predict the risk level of each patient as follows: Risk score = (−0.757) × exp (MYO1H) + (−0.31) × exp (TNFRSF13B) + (−0.235) × exp (GNG8) + 0.133 × exp (FOXA2) + 0.117 × exp (METTL7B) + 0.186 × exp (SYT14) (Figures 5E–H).

**FIGURE 6:**
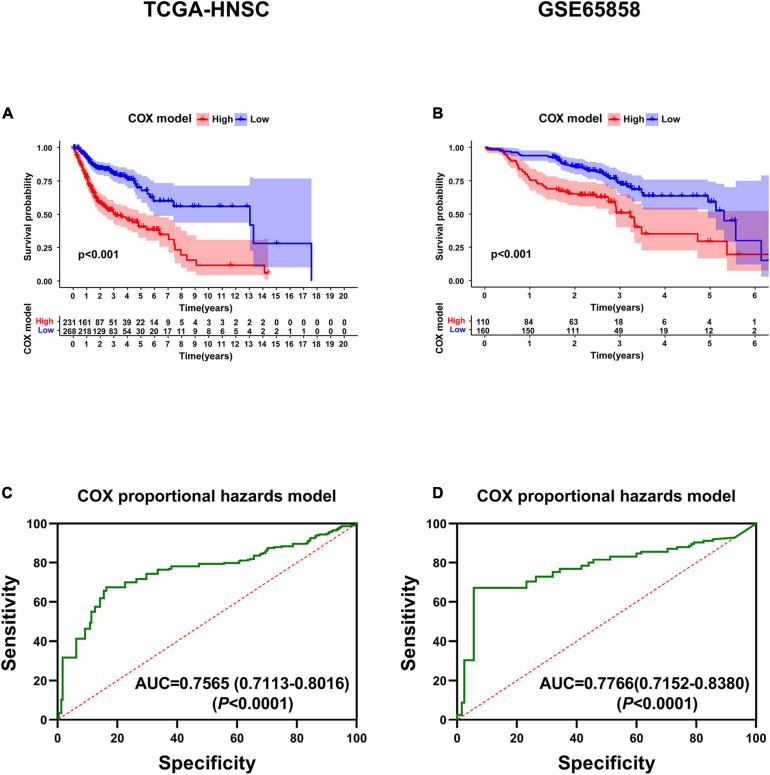
Validation of the six-gene prognostic signature. **(A)** Survival analysis of the prognostic signature in TCGA. **(B)** Survival analysis of the prognostic signature in GSE65858. **(C)** AUC in ROC analysis for the signature at overall survival in TCGA. **(D)** AUC in ROC analysis for the signature at overall survival in GSE65858.

KM survival analysis showed that patients with high-risk levels had significantly lower overall survival rates (Figures 6A,B), and the AUC of ROC was 0.7565 (*P* < 0.0001, 95%CI of HR: 0.7113–0.8016) in TCGA, and 0.7766 (*P* < 0.0001, 95%CI of HR: 0.7152–0.8380) in GSE65858 (Figures 6C,D), demonstrating that this risk score model is of a certain value. The chi-square test showed that, Age, location of tumor, tumor stage, T stage, N stage M stage and risk level are all significantly correlated with survival status (Table 2), among them, tumor stage, T stage, and N stage were also significantly correlated with risk level, it was worth noting that pathological grade is related to risk level, but not to clinical outcome (Table 3).

**TABLE 2:** The relationship between clinical outcome and clinical factors.

Category	Alive	Dead	Total	*P*-value
	(*n* = 333)	(*n* = 166)	(*n* = 499)	
**Age**	Mean 59.96 ± 11.13	Mean 63.31 ± 13.05		**0.020**
< 60	159 (31.9%)	61 (12.1%)	220 (44.0%)	
≥ 60	174 (34.9%)	105 (21.1%)	279 (56.0%)	
**Gender**				0.060
Male	253 (50.7%)	113 (22.7%)	366 (73.4%)	
Female	80 (16.0%)	53 (10.6%)	133 (26.6%)	
**Smoking**				0.114
Yes	248 (49.7%)	130 (26.1%)	378 (75.8%)	
No	81 (16.2%)	29 (5.8%)	110 (22.0%)	
Unknown	4 (0.8%)	7 (1.4%)	11 (2.2%)	
**Alcohol**				0.095
Yes	227 (45.5%)	103 (20.7%)	330 (66.2%)	
No	96 (19.2%)	61 (12.2%)	157 (31.4%)	
Unknown	10 (2.0%)	2 (0.4%)	12 (2.4%)	
**HPV status**				Invalid
Negative	70 (14.0%)	10 (2.0%)	80 (16.0%)	
Positive	31 (6.2%)	1 (0.2%)	32 (6.4%)	
Unknown	232 (46.5%)	155 (31.1%)	387 (77.6%)	
**Location of tumor**				**0.022**
Alveolar ridge	14 (2.8%)	4 (0.8%)	18 (3.6%)	
Base of tongue	18 (3.6%)	5 (1.0%)	23 (4.6%)	
Buccal mucosa	17 (3.4%)	5 (1.0%)	22 (4.4%)	
Floor of mouth	37 (7.4%)	23 (4.6%)	60 (12.0%)	
Hard palate	6 (1.2%)	1 (0.2%)	7 (1.4%)	
Hypopharynx	7 (1.4%)	3 (0.6%)	10 (2.0%)	
Larynx	72 (14.5%)	39 (7.8%)	111 (22.3%)	
Lip	2 (0.4%)	1 (0.2%)	3 (0.6%)	
Oral cavity	35 (7.0%)	37 (7.4%)	72 (14.4%)	
Oral tongue	85 (17.1%)	40 (8.0%)	125 (25.1%)	
Oropharynx	8 (1.6%)	1 (0.2%)	9 (1.8%)	
Tonsil	32 (6.4%)	7 (1.4%)	39 (7.8%)	
**Pathological grade**				0.578
G1	44 (8.8%)	17 (3.4%)	61 (12.2%)	
G2	198 (39.7%)	100 (20.0%)	298 (59.7%)	
G3	76 (15.2%)	42 (8.5%)	118 (23.7%)	
Unknown	15 (3.0%)	7 (1.4%)	22 (4.4%)	
**Tumor stage**				**0.004**
I + II	81 (16.2%)	22 (4.4%)	103 (20.6%)	
III + IV	252 (50.5%)	144 (28.9%)	396 (79.4%)	
**T stage**				**0.001**
T1 + T2	149 (29.9%)	48 (9.6%)	197 (39.5%)	
T3 + T4	184 (36.9%)	118 (23.6%)	302 (60.5%)	
**N stage**				**0.018**
N0	130 (26.1%)	47 (9.4%)	177 (35.5%)	
NX + N1 + N2 + N3	203 (40.7%)	119 (23.8%)	322 (64.5%)	
**M stage**				**0.015**
M0	281 (56.3%)	153 (30.7%)	434 (87.0%)	
MX + M1	52 (10.4%)	13 (2.6%)	65 (13.0%)	
**Risk level**				**<0.001**
Low	213 (42.7%)	55 (11.0%)	268 (53.7%)	
High	120 (24.0%)	111 (22.3%)	231 (46.3%)	

*P < 0.05 is indicated in bold.*

**TABLE 3:** The relationship between risk level and clinical factors.

Category	Low risk	High risk	Total	*P*-value
	(*n* = 268)	(*n* = 231)	(*n* = 499)	
**Age**	Mean 60.97 ± 11.58	Mean 61.20 ± 12.32		0.487
< 60	122 (24.4%)	98 (19.6%)	220 (44.0%)	
≥ 60	146 (29.3%)	133 (26.7%)	279 (56.0%)	
**Gender**				0.908
Male	196 (39.3%)	170 (34.1%)	366 (73.4%)	
Female	72 (14.4%)	61 (12.2%)	133 (26.6%)	
**Smoking**				0.058
Yes	195 (39.1%)	183(36.7%)	378 (75.8%)	
No	68 (13.6%)	42 (8.4%)	110 (22.0%)	
Unknown	5 (1.0%)	6 (1.2%)	11 (2.2%)	
**Alcohol**				0.677
Yes	179 (35.9%)	151 (30.3%)	330 (66.2%)	
No	82 (16.4%)	75 (15.0%)	157 (31.4%)	
Unknown	7 (1.4%)	5 (1.0%)	12 (2.4%)	
**HPV status**				Invalid
Negative	48 (9.6%)	32 (6.4%)	80 (16.0%)	
Positive	27 (5.4%)	5 (1.0%)	32 (6.4%)	
Unknown	193 (38.7%)	194 (38.9%)	387 (77.6%)	
**Location of tumor**				0.124
Alveolar ridge	11 (2.2%)	7 (1.4%)	18 (3.6%)	
Base of tongue	13 (2.6%)	10 (2.0%)	23 (4.6%)	
Buccal mucosa	8 (1.6%)	14 (2.8%)	22 (4.4%)	
Floor of mouth	31 (6.2%)	29 (5.8%)	60 (12.0%)	
Hard palate	5 (1.0%)	2 (0.4%)	7 (1.4%)	
Hypopharynx	5 (1.0%)	5 (1.0%)	10 (2.0%)	
Larynx	59 (11.8%)	52 (10.5%)	111 (22.3%)	
Lip	2 (0.4%)	1 (0.2%)	3 (0.6%)	
Oral cavity	38 (7.6%)	34 (6.8%)	72 (14.4%)	
Oral tongue	60 (12.0%)	65 (13.1%)	125 (25.1%)	
Oropharynx	5 (1.0%)	4 (0.8%)	9 (1.8%)	
Tonsil	31 (6.2%)	8 (1.6%)	39 (7.8%)	
**Pathological grade**				**0.016**
G1	41 (8.2%)	20 (6.6%)	61 (12.2%)	
G2	147 (29.5%)	151 (26.3%)	298 (59.7%)	
G3	70 (14.0%)	48 (11.1%)	118 (23.7%)	
Unknown	10 (2.0%)	12 (2.4%)	22 (4.4%)	
**Tumor stage**				**0.01**
I + II	67 (13.4%)	36 (7.2%)	103 (20.6%)	
III + IV	201 (40.3%)	195 (39.1%)	396 (79.4%)	
**T stage**				**0.005**
T1 + T2	121 (24.2%)	76 (15.2%)	197 (39.5%)	
T3 + T4	147 (29.5%)	155 (31.1%)	302 (60.5%)	
**N stage**				**0.005**
N0	110 (2.2%)	67 (13.4%)	177 (35.5%)	
NX + N1 + N2 + N3	158 (31.7%)	164 (32.9%)	322 (64.5%)	
**M stage**				0.577
M0	231 (46.3%)	203 (40.7%)	434 (87.0%)	
MX + M1	37 (74.1%)	28 (5.6%)	65 (13.0%)	

*P < 0.05 is indicated in bold.*

### Functional Enrichment Analysis of the Prognostic Gene Signature

GSEA analysis was performed to explore the related functional pathways involving the six-gene signature in HNSCC. It turned out that highly expressed FOXA2, SYT14, and METTL7B were mainly enriched in the ECM receptor interaction, TGFβ signaling pathway, WNT signaling pathway, and MAPK signaling pathway, respectively, while lowly expressed FOXA2, SYT14, and METTL7B were mainly correlated with oxidative phosphorylation, peroxisome, fatty acid metabolism, and Natural killer cell mediated cytotoxicity. Highly expressed MYO1H was mainly enriched in linoleic acid metabolism, metabolism of xenobiotics by cytochrome P450, and retinol metabolism, while lowly expressed MYO1H was mainly enriched in glycosaminoglycan biosynthesis chondroitin sulfate, P53 signaling pathway, and small cell lung cancer. Interestingly, the high expression of GNG8 and TNFRSF13B is not only associated with the VEGF signaling pathway and JAK-STAT signaling pathway, but also related to immune-related pathways, including antigen processing and presentation, B and T cell receptor signaling pathway, and toll-like receptor signaling pathway, while lowly expressed GNG8 and TNFRSF13B were enriched in the adherens junction and proteasome (Table 4). These results also explain, to a certain extent, why GNG8 and TNFRSF13B were highly expressed in the metastasis group, but correlated with poor prognosis.

**TABLE 4:** Single-gene GSEA in FOXA2, METTL7B, SYT14, GNG8, MYO1H, and TNFRSF13B.

Gene	High expression	Low expression
**FOXA2**	ECM receptor interaction	Fatty acid metabolism
	WNT signaling pathway	T cell receptor signaling pathway
	Focal adhesion	Oxidative phosphorylation
	TGFβ signaling pathway	Peroxisome
	Glycosaminoglycan biosynthesis chondroitin sulfata	Arachidonic acid metabolism
**METTL7B**	Basal cell carcinoma	Linoleic acid metabolism
	ECM receptor interaction	
	Lysosome	
	MAPK signaling pathway	
	WNT signaling pathway	
**SYT14**	Basal cell carcinoma	Fatty acid metabolism
	Pathways in cancer	Natural killer cell mediated cytotoxicity
	TGFβ signaling pathway	
	WNT signaling pathway	
**GNG8**	Antigen processing and presentation	Adherens junction
	B cell receptor signaling pathway	Tight junction
	Cytokine cytokine receptor interaction	
	JAK stat signaling pathway	
	Toll like receptor signaling pathway	
	VEGF signaling pathway	
**MYO1H**	Retinol metabolism	Cell cycle
	Glycosaminoglycan biosynthesis chondroitin sulfata	Linoleic acid metabolism
	Metabolism of xenobiotics by cytochrome P450	P53 signaling pathway
		Small cell lung cancer
		Cytokine cytokine receptor interaction
**TNFRSF13B**	Natural killer cell mediated cytotoxicity	Proteasome
	Primary immunodeficiency	
	T cell receptor signaling pathway	
	Toll like receptor signaling pathway	
	VEGF signaling pathway	

### Validation of the Prognostic Value of the Six-Gene Signature

**FIGURE 7:**
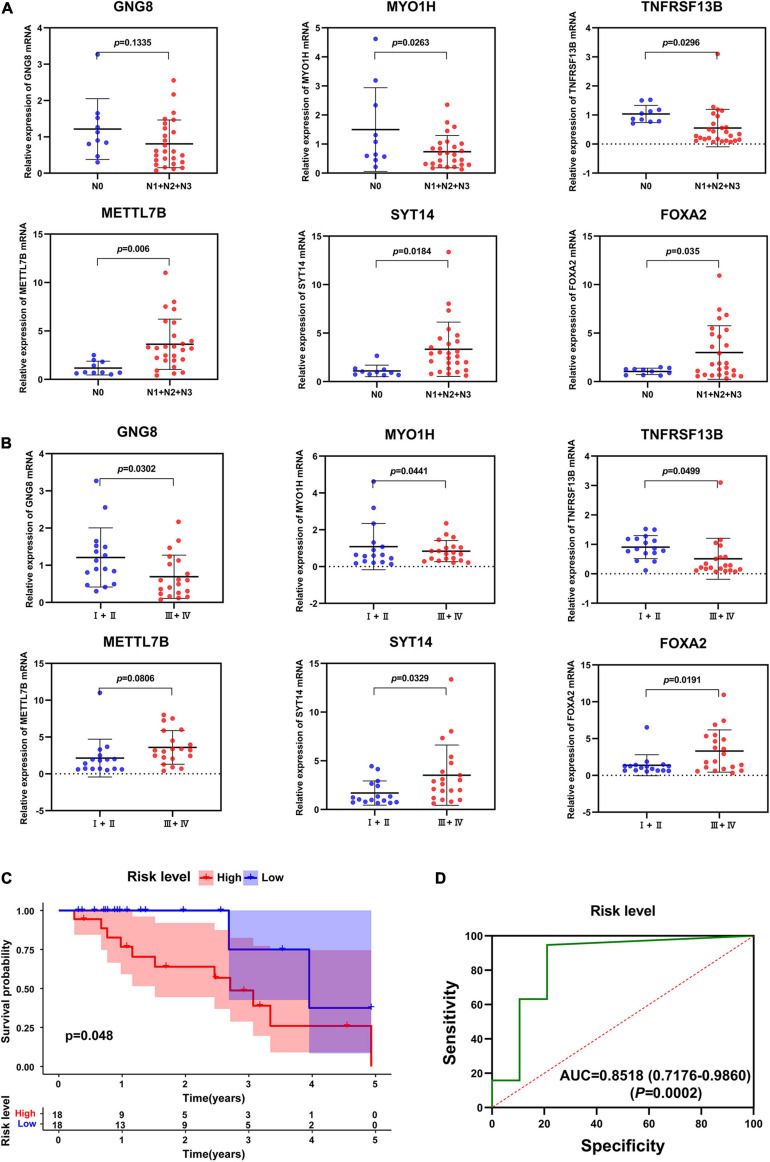
Validation of six-gene prognostic signature by qRT-PCT. **(A)** The expression of these 6 genes in different N stages. **(B)** The expression of these 6 genes in different tumor stages. **(C)** Survival analysis of the prognostic signature in clinical samples. **(D)** AUC in ROC analysis for the signature at overall survival in clinical samples.

Next, the mRNA expression of GNG8, MYO1H, TNFRSF13B, METTL7B, SYT14, and FOXA2 were detected in 36 HNSCC tissues by qRT-PCR. The results showed that the expression of MYO1H and TNFRSF13B was significantly down-regulated, while the expression of METTL7B, SYT14, and FOXA2 was significantly up-regulated in the HNSCC tissues with lymphatic metastasis (Figure 7A). Moreover, the expression of GNG8, MYO1H, and TNFRSF13B was significantly down-regulated, while the expression of SYT14 and FOXA2 was significantly up-regulated in the samples with higher tumor stage (Figure 7B). The risk scores of 36 patients were also calculated, and the high-risk group had a lower overall survival rate (Figure 7C). The AUC value of the ROC curve was 0.8515, indicating high accuracy of the six-gene signature in HNSCC samples (Figure 7D).

### The Correlation Between the Risk Level and Immune Cell Infiltration

**FIGURE 8:**
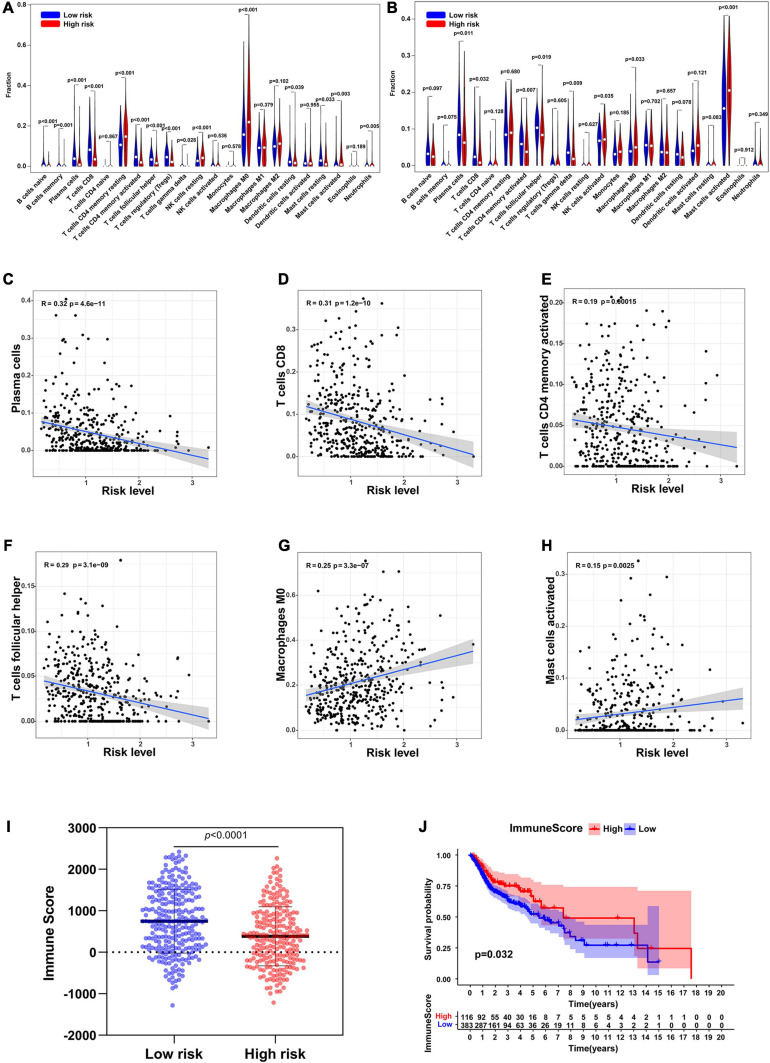
Immune cell infiltration in low-risk and high-risk group. **(A)** Infiltration of 22 types of immune cells in TCGA. **(B)** Infiltration of 22 types of immune cells in GSE65858. **(C–H)** The relationship between the risk level and the infiltration levels of plasma cells, T cells CD8, T cells CD4 memory activated, T cells follicular helper, Macrophages M0, and Mast cells activated. **(I)** Immune scores in low-risk and high-risk groups. **(J)** Survival analysis of the immune score.

The CIBERSORT analysis indicated that the infiltration levels of plasma cells, T cells CD8, T cells CD4 memory activated and T cells follicular helper were significantly lower in the high-risk group than those in the low-risk group, and were negatively associated with the risk level. The infiltration levels of macrophages M0 and mast cells activated in the low-risk group were significantly higher than those in the high-risk group, and were positively correlated with the risk level (Figures 8A–H). ESTIMATE algorithm showed that the immune score of the high-risk group was significantly lower than that of the low-risk group (Figure 8I). In addition, the immune score also significantly affected the overall survival rate of patients in TCGA (Figure 8J).

### Establishment and Validation of the Multi-Factor Prognostic Model

**FIGURE 9:**
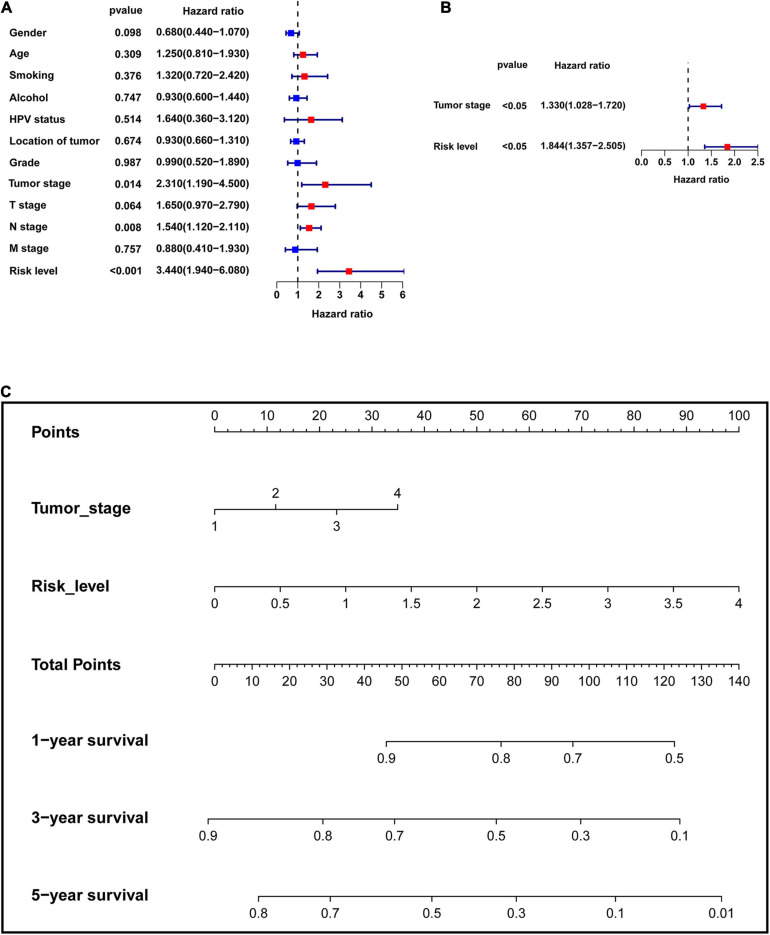
Establishment of the multi-factor prognostic model. **(A)** Univariate Cox proportional hazards regression analysis was performed to assess which factors have significant prognostic value. **(B)** Multivariate Cox proportional hazards regression analysis was used to establish the multivariate prognostic model. **(C)** Nomogram was built to predict 1-, 3-, and 5-year survival rate.

**FIGURE 10:**
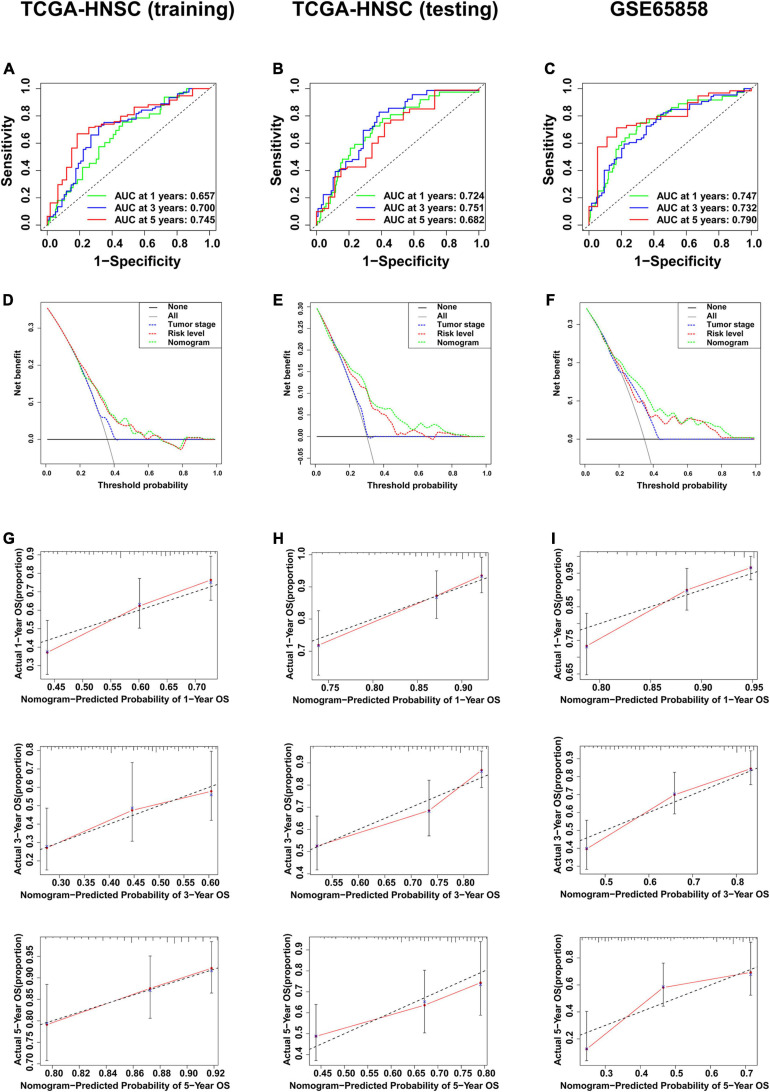
Validation of the multi-factor prognostic model. **(A)** AUC in ROC analysis for the prognostic model in TCGA training cohort. **(B)** AUC in ROC analysis for the prognostic model in TCGA testing cohort. **(C)** AUC in ROC analysis for the prognostic model in GSE65858. **(D)** DCA for the prognostic model in TCGA training cohort. **(E)** DCA for the prognostic model in TCGA testing cohort. **(F)** DCA for the prognostic model in GSE65858. **(G)** Calibration plot for 1-, 3-, and 5-year overall survival in TCGA training cohort. **(H)** Calibration plot for 1-, 3-, and 5-year overall survival in TCGA testing cohort. **(I)** Calibration plot for 1-, 3-, and 5-year overall survival in GSE65858.

The clinical data of the training, testing cohorts and GSE65858 are shown in Table 5. Through univariate Cox proportional hazards regression analysis, we found that tumor stage, N stage, and risk level were all high-risk factors for prognosis in the training cohort (*P* < 0.05) (Figure 9A), showing that the tumor stage, N stage, and risk level all had prognostic value. The three factors were then included in multivariate Cox proportional regression analysis. We found that the tumor stage and risk level were finally selected, indicating that they could be utilized as independent factors to predict the clinical outcome of patients with HNSCC (*P* < 0.05) (Figure 9B). Further, a nomogram was built with the two factors to predict the 1-, 3-, and 5-year survival rates (Figure 9C). In the training cohort, the AUC values of the ROC curve for the nomogram were 0.657, 0.700, and 0.745, at 1-, 3-, and 5-year survival, respectively, and 0.724, 0.751, and 0.682 in the testing cohort, 0.747, 0.732, and 0.790 in GSE65858 (Figures 10A–C). DCA demonstrated that the combined model consisting of risk level and tumor stage showed the best clinical net benefit (Figures 10D–F). The calibration plot also showed the accuracy of this nomogram in the three cohorts, and the C-index values of the nomogram were 0.656, 0.7065, and 0.7055 in the training, testing cohorts and GSE65858, respectively (Figures 10G–I).

**TABLE 5:** Clinical data of TCGA-HNSC, GSE65858, and clinical samples.

Category	TCGA training	TCGA testing	GSE65858	Clinical samples
**Age**	Mean = 61.5 ± 11.0	Mean = 60.7 ± 12.8	Mean = 60.1 ± 10.3	Mean = 63.4 ± 12.2
< 60	105 (21.0%)	115 (23.0%)	153 (56.7%)	21 (58.3%)
≥ 60	145 (29.1%)	134 (26.9%)	117 (43.3%)	15 (41.7%)
**Gender**				
Male	189 (37.9%)	177 (35.5%)	223 (82.6%)	27 (75.0%)
Female	61 (12.2%)	72 (14.4%)	47 (17.4%)	9 (25.0%)
**Smoking**				
Yes	46 (9.2%)	64 (12.8%)	48 (17.8%)	0
No	198 (6.6%)	180 (7.8%)	222 (82.2%)	0
Unknown	6 (1.2%)	5 (1.0%)	0 (0%)	36 (100%)
**Alcohol**				
Yes	169 (33.9%)	161 (32.3%)	239 (88.5%)	0
No	75 (15.0%)	82 (16.4%)	31 (11.5%)	0
Unknown	6 (1.2%)	6 (1.2%)	0 (0%)	36 (100%)
**HPV status**				
Negative	40 (8.0%)	40 (8.0%)	196 (72.6%)	0
Positive	16 (3.2%)	16 (3.2%)	74 (27.4%)	0
Unknown	194 (38.9%)	193 (38.7%)	0	36 (100%)
**Location of tumor**				
Alveolar ridge	8 (1.6%)	10 (2.0%)	0	0
Base of tongue	14 (2.8%)	9 (1.8%)	0	2 (5.6%)
Buccal mucosa	8 (1.6%)	14 (2.8%)	0	2 (5.6%)
Floor of mouth	29 (5.8%)	31 (6.2%)	0	0
Hard palate	0	7 (1.4%)	0	0
Hypopharynx	7 (1.4%)	3 (0.6%)	33 (12.2%)	0
Larynx	59 (11.9%)	52 (10.4%)	48 (17.8%)	0
Lip	1 (0.2%)	2 (0.4%)	0	0
Oral cavity	43 (8.6%)	29 (5.8%)	87 (32.2%)	22 (61.1%)
Oral tongue	60 (12.0%)	65 (13.1%)	0	10 (27.7%)
Oropharynx	4 (0.8%)	5 (1.0%)	102 (37.8%)	0
Tonsil	17 (3.4%)	22 (4.4%)	0	0
**Pathological grade**				
G1	30 (6.0%)	31 (6.2%)	0	11 (30.6%)
G2	153 (30.7%)	145 (29.1%)	0	14 (38.8%)
G3	55 (11.0%)	63 (12.6%)	0	11 (30.6%)
Unknown	12 (2.4%)	10 (2.0%)	270 (100%)	0
**Tumor stage**				
I + II	49 (9.8%)	54 (10.8%)	55 (20.4%)	16 (44.4%)
III + IV	201 (40.3%)	195 (39.1%)	215 (79.6%)	20 (55.6%)
**T stage**				
T1 + T2	96 (19.2%)	101 (20.2%)	115 (42.6%)	16 (44.4%)
T3 + T4	154 (30.9%)	148 (29.7%)	155 (57.4%)	20 (55.6%)
**N stage**				
N0	86 (17.2%)	91 (18.2%)	94 (34.8%)	10 (27.8%)
NX + N1 + N2 + N3	164 (32.9%)	158 (31.7%)	176 (65.2%)	26 (72.2%)
**M stage**				
M0	217 (43.5%)	217 (43.5%)	263 (97.4%)	36 (100%)
MX + M1	33 (6.6%)	32 (6.4%)	7 (2.6%)	0
**Status**				
Alive	160 (32.1%)	173 (34.7%)	176 (65.2%)	23 (63.9%)
Dead	90 (18.0%)	76 (15.2%)	94 (34.8%)	13 (36.1%)

## Discussion

Because of the special anatomical features of the head and neck, HNSCC is very prone to early metastasis, especially lymphatic metastasis, which seriously affects the patient’s prognosis (Evans et al., 2019). Exploring molecular biological markers is of great significance to an early diagnosis, prognosis prediction, and treatment strategies of HNSCC. Studies based on deep sequencing have revealed various biomarkers for HNSCC diagnosis. A previous study has developed a 16-gene prognostic signature that can predict the prognosis of patients with tongue squamous cell carcinoma, including CD96, HNF1B, and SMG1 (Qiu et al., 2017). VEGF overexpression is closely related to advanced disease and poor survival in *in vitro* experiments and bioinformatics, revealing a novel HDGF/HIF-1α/VEGF axis in oral cancer prognosis (Lin et al., 2019). TGFBI, SPP1, and LAMB3 have been identified as potential biomarkers and survival-influencing factors of HNSCC (Shen et al., 2019). Using the TCGA data, Zhou et al. (2018) found that FAM135B methylation is a favorable independent prognostic biomarker for the overall survival of patients with HNSCC. In addition, Zhang et al. (2020) have identified 14 genes related to immune cell infiltration and can predict the prognosis of HNSCC. However, most studies are limited to comparing non-tumor and tumor tissues, whether these gene signatures are related to the metastasis-prone characteristics of HNSCC is little available. Therefore, exploring biomarkers closely related to HNSCC metastasis and constructing a comprehensive prognostic prediction model are extremely important for improving the survival rate of HNSCC patients.

In this study, we explored DEGs between the metastatic samples and the non-metastatic samples in order to obtain biomarkers related to metastasis. For more reliability of the results, we obtained 108 common DEGs from different data sets, which were likely to be changed in the majority of HNSCC samples. GO analysis showed that keratinization, muscle filament sliding and actin binding were mainly enriched in the “biological process” and “molecular function,” which is consistent with a previous study which found that highly invasive tumor cells exhibit enhanced actin polymerization activity and abnormal expression of actin regulatory proteins (Bravo-Cordero et al., 2012). In the “cellular component,” extracellular exosomes and extracellular spaces were significantly enriched, which showed that the communication between cells might be essential for cancer progression. In KEGG and GSEA analyses, a variety of signaling pathways were enriched. The P53 signaling pathway, cell cycle, Ras signaling pathway, and VEGF signaling pathway have been proven to play key roles in the progression of many tumors (Diez et al., 2011; Sohn et al., 2018; Meireles Da Costa et al., 2020). In general, the functions and pathways enriched in this study might also promote HNSCC metastasis.

As shown in the KM analysis of the 108 common DEGs, 12 DEGs significantly affected the overall survival rate. Next, we conducted Cox proportional hazards regression analysis on these 12 DEGs, and a six-gene prognostic signature was constructed, including GNG8, MYO1H, TNFRSF13B, SYT14, METTL7B, and FOXA2. The risk score of each patient was calculated, KM analysis showed that the high-risk group had significantly worse overall survival than the low-risk group. To further verify the reliability of this six-gene signature, we collected 36 HNSCC tissue samples and detected the mRNA expression of the six genes by qRT-PCR, and got similar results. The results showed that the expression of MYO1H and TNFRSF13B was significantly down-regulated, while the expression of METTL7B, SYT14, and FOXA2 was significantly up-regulated in the HNSCC tissues with lymphatic metastasis. The expression of GNG8, MYO1H, and TNFRSF13B was significantly down-regulated, while the expression of SYT14 and FOXA2 was significantly up-regulated in the samples with higher tumor stage, the samples with high risk had lower overall survival rates.

Current research shows that MYO1H is significantly related to mandibular deformities (Tassopoulou-Fishell et al., 2012). Interestingly, as the entire myosin superfamily, its internal members have different roles in tumors; for example, MYO1A can inhibit gastrointestinal tumors (Mazzolini et al., 2013), while MYO1E can promote breast cancer invasion (Hallett et al., 2012). It is worth noting that compared with the non-metastasis group, the expression of MYO1H was lower in the metastasis group. The GSEA results also showed that the low expression of MYO1H was significantly related to the cell cycle and P53 signaling pathway.

TNFRSF13B codes for the transmembrane activator, calcium modulator, and cyclophilin ligand interactor (TACI), which is mainly expressed on the surface of several B cells and in the marrow of humans (Sakurai et al., 2007). As the main receptor of B cell activation factor (BAFF), TACI can be combined with BAFF to trigger the NFκB typical signal pathway, thereby activating the NFκB anti-apoptotic cascade (Ouyang et al., 2012). TCAI is also a proliferation-inducing ligand (APRIL), and plays an important role in tumor growth and metastasis, including the promotion of cell cycle proliferation and anti-apoptosis (Garcia-Castro et al., 2015). Such a mechanism was verified in a study of breast cancer (Abo-Elfadl et al., 2020). Our results also showed that TNFRSF13B was highly expressed in the metastasis group; unexpectedly, it was low in the high-risk group. GSEA results showed that high TNFRSF13B expression is significantly related to the VEGF signaling pathway, which promotes metastasis, but is also associated with immune responses related to tumors, promoting a good prognosis for patients with HNSCC.

A recent study showed that GNG8 could regulate cognitive function by regulating cholinergic activity (Lee et al., 2020). However, there are few reports about this gene in tumors. The recurrence of sporadic chronic lymphocytic leukemia (CLL) and small lymphocytic lymphoma (SLL) may be related to the signaling pathways of certain G proteins (including GNG8) and G protein-coupled receptors (Nuckel et al., 2003). Moreover, there is also a certain relationship between GNG8 and the migration of CLL and SLL cells. In this study, we found that the high expression of GNG8 is significantly related to the VEGF and JAK-STAT signaling pathways, but similarly, it may also cause corresponding immune responses.

It has been reported that FOXA2 is highly expressed in colorectal cancer, participates in the epithelial–mesenchymal transition (EMT) process, and is closely related to the metastasis and clinical staging of colorectal cancer (Wang et al., 2018). In addition, FOXA2 has also been shown to be a target gene of MiR-942, which is involved in the proliferation, migration, and invasion of breast cancer cells (Zhang J. et al., 2019). Through the analysis of our data, we found that FOXA2 was highly expressed in the metastasis and high-risk groups, and was significantly related to the TGFβ signaling pathway and WNT signaling pathway.

METTL7B, encoded by the gene with the same name, is a member of the METTL protein family. The members of this protein family are DNA, RNA, and protein methyltransferases (Ignatova et al., 2019). In the study of non-small cell lung cancer (NSCLC), METTL7B is the target of NSCLC treatment and is involved in the regulation of the tumor cell cycle (Liu et al., 2020). In addition, METTL7B may also activate TGFβ1 and induce EMT in thyroid cancer (Ye et al., 2019). Our results also showed that METTL7B is highly expressed in both the metastasis group and the high-risk group, and is related to signaling pathways, such as the WNT and MAPK signaling pathways.

The function of SYT14 in human cancer is unclear, it has been reported that RNAi-mediated SYT14 knockdown inhibits the growth of human glioma cell line (Sheng et al., 2018). Our results showed thatSYT14 is also highly expressed in both the metastasis group and the high-risk group, and is highly related to the pathways in cancer, TGFβ signaling pathway and WNT signaling pathway.

Immune cell infiltration is an important part of the tumor microenvironment and has important value in predicting tumor prognosis (Gentles et al., 2015). In this study, we found that patients with low immune scores had worse prognosis, and our six-gene signature was significantly related to immune cell infiltration, plasma cells. T cells CD8, T cells CD4 memory activated, and T cells follicular helper were negatively correlated with risk level, while macrophages M0 and mast cells activated were positively correlated with risk level. plasma cells, T cells CD8, T cells CD4 memory activated, and T cells follicular helper were reported to play important roles in inhibiting tumors (Kim and Cantor, 2014; Wouters and Nelson, 2018; Panneton et al., 2019), while macrophages and mast cells activated have been proved to be associated with tumor metastasis and poor prognosis (Komohara et al., 2016; Zhang et al., 2020).

Moreover, we found that tumor stage, T stage, and N stage were all significantly related to survival status and risk level based on the six-gene signature in the chi-square test. As shown in the univariate Cox analysis, tumor stage, N stage, and risk level are all poor prognostic indicators of HNSCC. As the traditional tumor stage has certain limitations in predicting the prognosis of HNSCC (Beltz et al., 2018), we established a prognostic model that combines six-gene signatures related to metastasis with tumor stage in HNSCC using multivariate Cox analysis. Next, the nomogram constructed based on this model provided a more intuitive evaluation tool. In the training cohort, the internal verification cohort and external verification cohort, the ROC curve, C-index, and calibration plot all revealed that the nomogram had ideal predictive performance in terms of the prognosis of 1-, 3-, and 5-year survival. As expected, the DCA also confirmed that the nomogram had higher net benefit than the traditional tumor stage. In addition, this prognostic model was developed based on metastasis, which was more in line with the characteristics of easy metastasis of HNSCC.

However, because of the nature of the data source, clinical data relative to treatment, epidemiology, etc. is limited or not available, the information about impact of tumor location and HPV status on the prognosis of HNSCC, and whether the effects of immunotherapy and chemotherapy are related to risk scores are limited in the current study. In addition, tumor is a very complex disease, including genetic and epigenetic changes which may all lead to inconsistencies in the prediction results. Therefore, it is necessary to combine with a large number of clinical samples for further verification.

In conclusion, we identified 108 DEGs related to HNSCC metastasis, and constructed a biological signature composed of GNG8, MYO1H, TNFRSF13B, METTL7B, SYT14, and FOXA2 for predicting the prognosis of patients with HNSCC. This biological signature was not only related to metastasis and prognosis, but also related to immune cell infiltration. The combined application of these biomarkers can divide HNSCC patients into low-risk or high-risk groups, which can provide useful guidance for individualized and precise treatment. Moreover, a multi-factor prognostic model integrating tumor stage and molecular biomarkers has been established, which can be an effective and convenient tool for the clinical prediction of HNSCC prognosis and selection of treatment strategies.

## Data Availability Statement

The original contributions presented in the study are included in the article/supplementary material, further inquiries can be directed to the corresponding author/s.

## Ethics Statement

The studies involving human participants were reviewed and approved by the Research Ethics Committee of the Beijing Stomatological Hospital of Capital Medical University (Approval No. CMUSH-IRB-KJ-PJ-2018-01). The patients/participants provided their written informed consent to participate in this study.

## Author Contributions

YS conducted the most of the data mining and data analysis, and drafted the most original manuscript. LL contributed to the design of the research and revised the manuscript. YL participated in data interpretation and manuscript revision. MZ contributed to the revision of the manuscript. XH contributed to the conception and analysis of the study. XT received research funding and participated in the design and supervision of the research. All authors reviewed the manuscript and allowed publication.

## Conflict of Interest

The authors declare that the research was conducted in the absence of any commercial or financial relationships that could be construed as a potential conflict of interest.

## Footnotes

1 https://portal.gdc.ancer.gov/

2 http://www.ncbi.nlm.nih.gov/geo/

3 https://www.gsea-msigdb.org/gsea/index.jsp
